# Discovery and validation of potential urinary biomarkers for bladder cancer diagnosis using a pseudotargeted GC-MS metabolomics method

**DOI:** 10.18632/oncotarget.14988

**Published:** 2017-02-01

**Authors:** Yang Zhou, Ruixiang Song, Chong Ma, Lina Zhou, Xinyu Liu, Peiyuan Yin, Zhensheng Zhang, Yinghao Sun, Chuanliang Xu, Xin Lu, Guowang Xu

**Affiliations:** ^1^ Key Laboratory of Separation Science for Analytical Chemistry, Dalian Institute of Chemical Physics, Chinese Academy of Sciences, Dalian 116023, China; ^2^ University of Chinese Academy of Sciences, Beijing 100049, China; ^3^ Department of Urology, Shanghai Changhai Hospital, Secondary Military Medical University, Shanghai 200433, China

**Keywords:** metabolomics, gas chromatography-mass spectrometry, biomarker, urine, bladder cancer

## Abstract

Bladder cancer (BC) is the second most prevalent malignancy in the urinary system and is associated with significant mortality; thus, there is an urgent need for novel noninvasive diagnostic biomarkers. A urinary pseudotargeted method based on gas chromatography–mass spectrometry was developed and validated for a BC metabolomics study. The method exhibited good repeatability, intraday and interday precision, linearity and metabolome coverage. A total of 76 differential metabolites were defined in the discovery sample set, 58 of which were verified using an independent validation urine set. The verified differential metabolites revealed that energy metabolism, anabolic metabolism and cell redox states were disordered in BC. Based on a binary logistic regression analysis, a four-biomarker panel was defined for the diagnosis of BC. The area under the receiving operator characteristic curve was 0.885 with 88.0% sensitivity and 85.7% specificity in the discovery set and 0.804 with 78.0% sensitivity and 70.3% specificity in the validation set. The combinatorial biomarker panel was also useful for the early diagnosis of BC. This approach can be used to discriminate non-muscle invasive and low-grade BCs from healthy controls with satisfactory sensitivity and specificity. The results show that the developed urinary metabolomics method can be employed to effectively screen noninvasive biomarkers.

## INTRODUCTION

Bladder cancer (BC) is the second most prevalent malignancy in the urinary system [1] and is associated with significant mortality worldwide [2]. BC tumorigenesis is related to genetic susceptibility, environmental exposure, and unhealthy lifestyles [3]. Early detection and treatment are effective methods for improving the five-year survival rate, up to 90% for non-muscle invasive (NMI) BC [4]. Current BC diagnoses are primarily based on urinary cytology and cystoscopy. However, the diagnostic sensitivity of urinary cytology is low, and cystoscopy is invasive and costly [5]. Hence, there is an urgent need to find new noninvasive, inexpensive biomarkers with high sensitivity and specificity for the diagnosis of BC.

Metabolomics is a powerful tool for investigating the variation of endogenous small molecules during life activities [6]. This method has also been used to study BC [7, 8], especially to identify biomarkers [9–11]. Huang et al. [9] found that a combined urinary biomarker composed of carnitine C9:1 and an unknown metabolite had high sensitivity and specificity in discriminating 27 BC patients from 32 healthy controls (HCs), although no validation was performed. Jin et al. [11] examined the urinary metabolic profiles of 138 BCs and 121 controls using liquid chromatography-mass spectrometry, and discovered that an orthogonal partial least-squares discriminant analysis (OPLS-DA) model based on metabolic profiling was appropriate for distinguishing BCs from controls. However, the lack of external validation and discrimination for early-stage BCs limits this model's significance in clinical application. Pasikanti et al. [12] performed urinary BC research with comprehensive two-dimensional gas chromatography-time-of-flight mass spectrometry. An OPLS-DA model based on metabolic profiling was used to discriminate BC from non-BC subjects, and the model was validated with 7 BC and 10 non-BC samples. Current urinary BC metabolomics studies have made promising progress, but still have some shortcomings, such as a lack of validation or limited validation subjects and a scarcity of early-diagnosis BC biomarkers.

Gas chromatography–mass spectrometry (GC-MS) is a popular method in metabolomics due to its high sensitivity and repeatability [13], available software for peak deconvolution [14], and commercial mass spectral libraries for identification [15]. This approach aims to analyze volatile and semi-volatile components using a nontargeted method in full scan mode or a targeted method in selected ion monitoring (SIM) mode. The nontargeted method displays wide metabolome coverage with limited sensitivity and linearity and complicated peak alignment [16, 17]. In contrast, the targeted method shows high sensitivity and accuracy with the detection of a few known compounds. By combining the advantages of both methods, a pseudotargeted method was proposed to analyze all detectable components in SIM mode [18]. This method has been used for various metabolomics studies [19, 20]. Urine is a noninvasive and readily available sample that is especially suitable for BC biomarker screening [21]. However, to date, a GC-MS-based urinary pseudotargeted method has not been developed.

**Figure 1:**
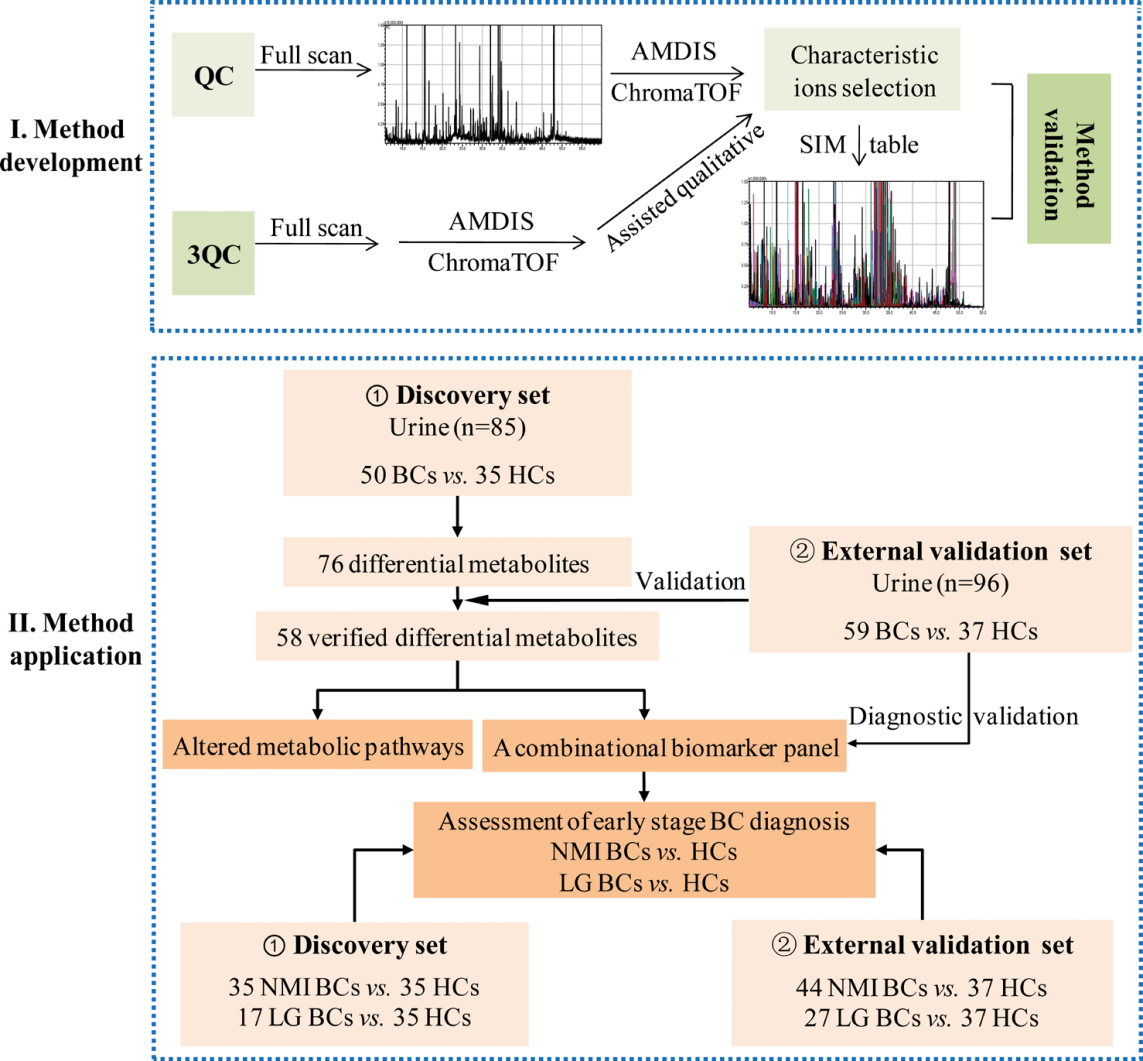
Experimental flow diagram

In this study, a GC-MS-based urinary pseudotargeted method was developed and applied for BC urinary metabolic profiling. A discovery urine set with 85 subjects (50 BCs and 35 HCs) and an independent validation set with 96 subjects (59 BCs and 37 HCs) were used to discover and verify the differential metabolites. A combinatorial biomarker panel was defined for BC and early-stage BC diagnosis. A flow diagram for this study is shown in Figure 1.

## RESULTS AND DISCUSSION

### Development and validation of the urinary pseudotargeted GC-MS metabolomics method

A urinary pseudotargeted GC-MS metabolomics method was developed based on GC-MS-SIM after peak detection, deconvolution and characteristic ion selection using quality control (QC) samples. The SIM acquisition table consisted of 28 groups including 465 characteristic ions. An example of the characteristic ion selection is shown in Supplementary Figure 1. A peak at 22.2 min in the QC sample was identified as a co-eluted peak after peak detection and deconvolution. The characteristic ions were successfully selected for the co-eluted peak using in-house software and chromTOF 4.43 (LECO, USA). The signal to noise ratio (S/N) of the co-eluted peaks was 430.3 and 135.6 with a typical Gaussian peak shape using the pseudotargeted method. In contrast, the S/N of the co-eluted peaks was 57.9 and 39.5, respectively, using the nontargeted method.

**Figure 2:**
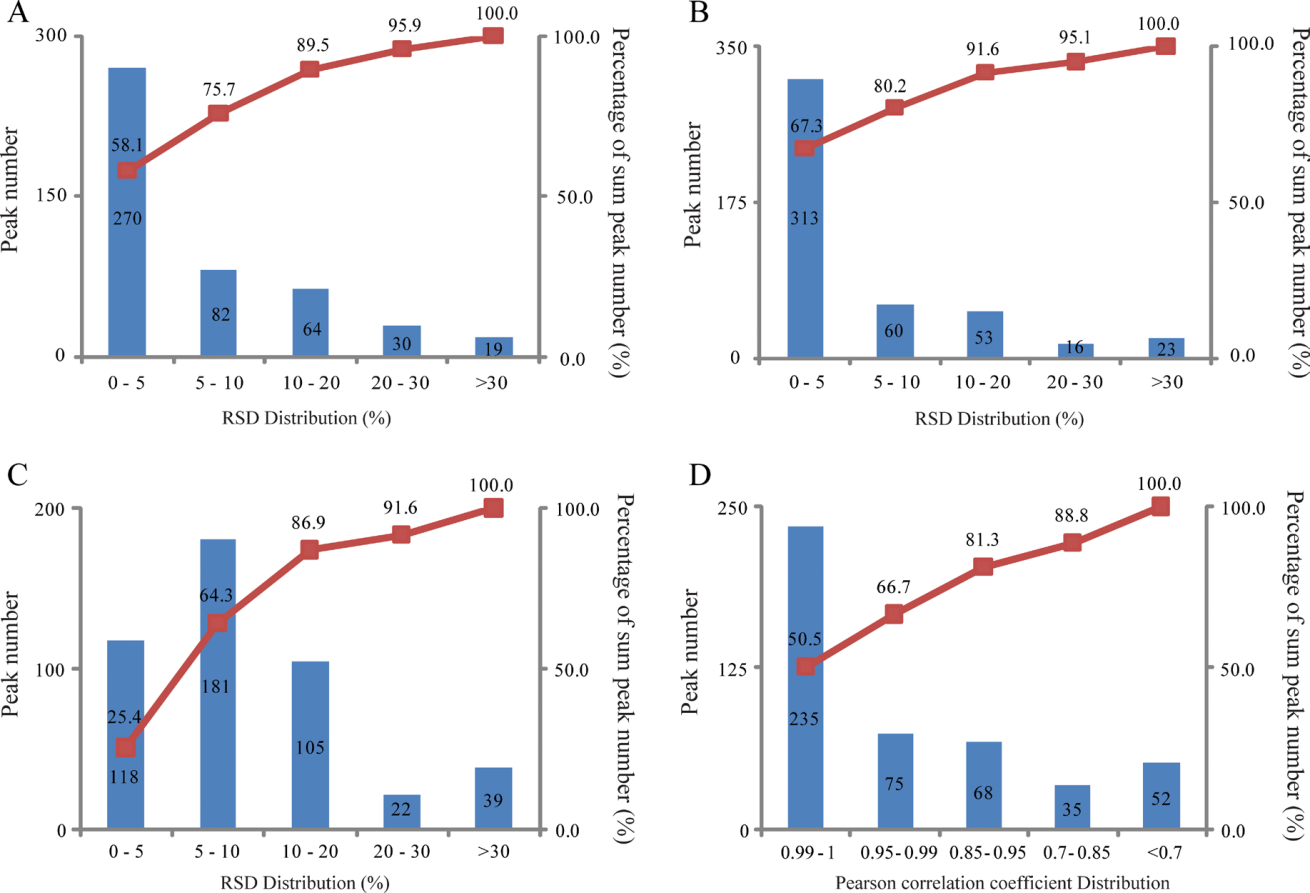
Method validation (**A**) Repeatability (*n* = 6). (**B**) Intraday precision (*n* = 6). (**C**) Interday precision (*n* = 2, 5 days). (**D**) Linearity.

The developed urinary pseudotargeted method was validated for its repeatability, intraday and interday precision and linearity. The repeatability was evaluated and expressed by the relative standard deviation (RSD) distribution; 75.7, 89.5 and 95.9% of the peaks had RSDs lower than 10, 20 and 30%, respectively (Figure 2A). The intraday precision was assessed by a QC sample analyzed six times, where 80.2, 91.6 and 95.1% of the peaks had RSDs lower than 10, 20 and 30%, respectively (Figure 2B). The interday precision was determined by analyzing two QC samples for five days, where 64.3, 86.9 and 91.6% of the peaks had RSDs lower than 10, 20 and 30%, respectively (Figure 2C). The linearity was expressed by the Pearson correlation coefficient for the peak intensity and metabolite concentrations. Among the peaks, 50.5, 66.7, 81.3 and 88.8% had a Pearson correlation coefficient higher than 0.99, 0.95, 0.85 and 0.7, respectively (Figure 2D). These results illustrate that the repeatability, intraday and interday precision, and linearity of the developed urinary pseudotargeted method were suitable for metabolic profiling analysis.

### Urinary metabolic profiling analysis of BC

The developed urinary pseudotargeted method was applied to BC metabolic profiling analysis. Score scatter plots from principal component analysis (PCA) showed that the QC samples were closely clustered (Supplementary Figure 2A ), and the Pearson correlation of any two QC samples was within 0.995–1.0 (Supplementary Figure 2B). The results illustrate that the data quality of the BC urinary metabolic profiling was good.

For metabolite identification, a 3-fold volume QC sample was used to enhance the peak strength of the low-abundance metabolites; thus, 42 low-content metabolites that were not identified in normal QC samples were successfully annotated. Finally, 231 metabolites involved in 57 metabolic pathways were annotated (Supplementary Table 1), including 84 organic acids and fatty acids, 63 saccharides and derivatives, 38 amino acids and derivatives, 13 nucleosides and derivatives, 9 alcohols, 8 phenols, 5 amines, 4 esters and 2 steroids. Among these, 147 metabolites were verified using standards.

**Figure 3:**
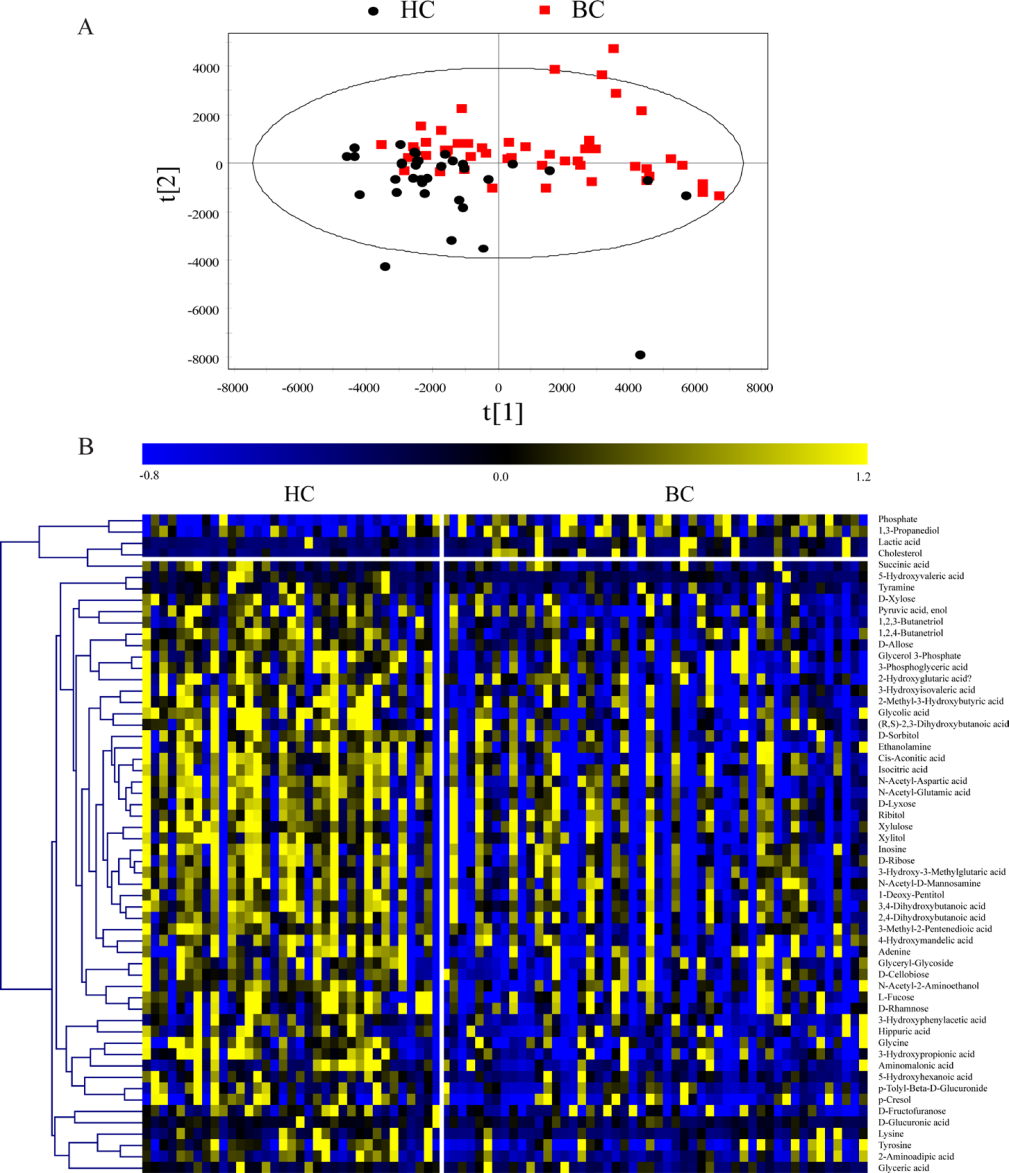
(**A**) Score scatter plots of the PLS-DA model with Pareto scaling. (**B**) Heat map of verified differential metabolites.

The 218 annotated metabolites with RSDs below 30% in the QC samples were used for further data analysis. To overview the differences in urinary metabolic profiling between the BC and HC groups, partial least-squares discriminant analysis (PLS-DA) with Pareto scaling was performed. Score scatter plots of the PLS-DA model showed that the BC group was clearly separated from the HC group in the second principal component (Figure 3A). The model was verified using a permutation test with 99 cycles. The R^2^Y and Q^2^Y intercept values were 0.289 and –0.136, respectively, suggesting that the PLS-DA model had no overfitting.

### Discovery and validation of differential metabolites

The Mann–Whitney *U* test and false discovery rate (FDR) correction were used to identify the differential metabolites (*p* < 0.05 and FDR < 0.15). In total, 76 differential metabolites related to BC were found in the discovery set. To validate the differential metabolites, independent batch urine samples (59 BC subjects and 37 age- and sex-matched HC subjects) were analyzed using the same procedures employed for the discovery set; 58 differential metabolites were validated with the same changing trends in the discovery set. Detailed information is shown in Supplementary Table 2. The relative contents of the verified differential metabolites in the discovery set are displayed in a heat map (Figure 3B). Most differential metabolite levels were significantly decreased in the BC group, including saccharides (e.g., D-ribose, D-glucuronic acid, D-lyxose, D-xylose, ribitol, xylitol, xylulose, D-cellobiose, D-rhamnose, L-fucose, D-allose, D-sorbitol, and N-acetyl-D-mannosamine), organic acids (e.g., 3-phosphoglyceric acid, isocitric acid, cis-aconitic acid, succinic acid, 2-hydroxyglutaric acid, 3-hydroxypropionic acid, 5-hydroxyvaleric acid, and 5-hydroxyhexanoic acid), amino acids (e.g., N-acetyl-aspartic acid, N-acetyl-glutamic acid, tyrosine, tyramine, glycine, lysine, and 2-aminoadipic acid) and nucleotides (e.g., adenine and inosine). In contrast, cholesterol, lactic acid, and 1, 3-propanediol levels were significantly increased in the BC group.

### Significantly altered metabolic pathways between BC and HC

**Figure 4:**
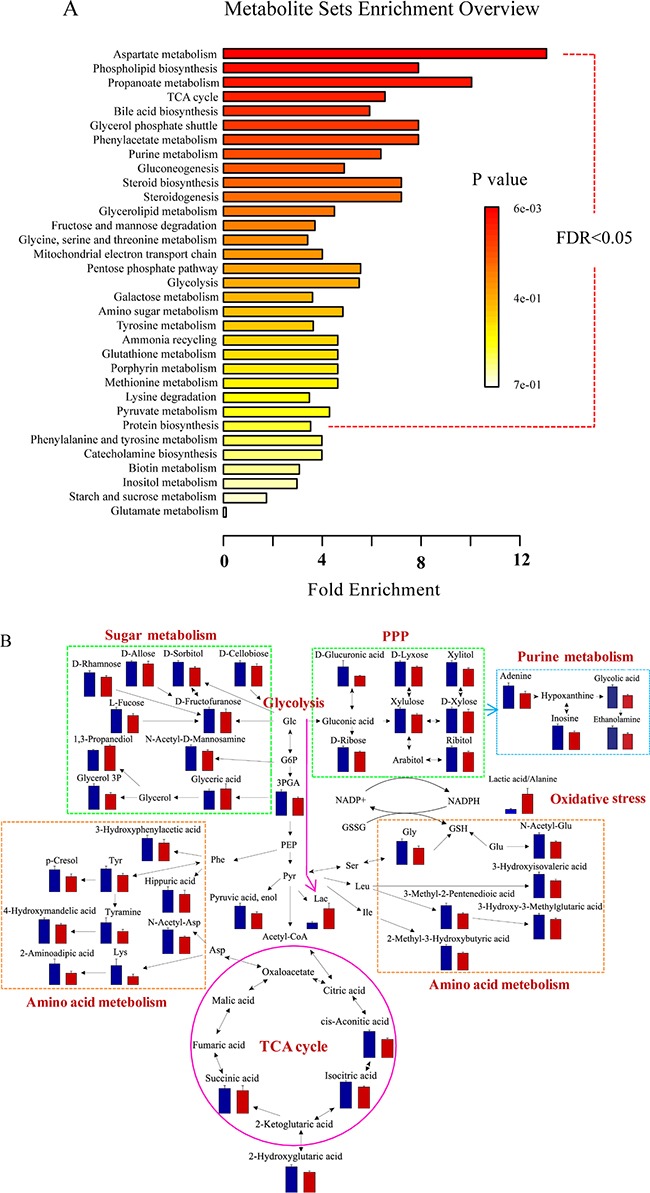
Differential metabolite pathway analysis (**A**) The globally altered pathways in BC. The color depth and column length indicate the disturbance degree of the pathway. (**B**) Pathway map of the significantly differential metabolites (*p* < 0.05) between BCs and HCs. The blue and red histograms denote the relative contents of the differential metabolites in the HCs and BCs, respectively. Abbreviations: glucose (Glc), glucose 6-phosphate (G6P), 3-phosphoglyceric acid (3PGA), phosphoenolpyruvic acid (PEP), pyruvic acid (Pyr), lactic acid (Lac), glycerol 3-phosphate (glycerol 3P), phenylalanine (Phe), aspartic acid (Asp), N-acetyl-aspartic acid (N-Acetyl-Asp), lysine (Lys), tyrosine (Tyr), serine (Ser), leucine (Leu), isoleucine (Ile), glycine (Gly), glutamic acid (Glu), N-acetyl-glutamic acid (N-Acetyl-Glu), glutathione (GSH), and oxidized glutathione (GSSH).

To determine the globally altered metabolic pathways induced by BC, pathway enrichment analysis was performed based on the validated differential metabolites in the discovery set (Figure 4A). Twenty-seven significantly changed pathways were found with an FDR < 0.05, including energy metabolism (e.g., glycolysis and tricarboxylic acid (TCA) cycle), amino acid metabolism (e.g., glycine, serine and threonine metabolism, tyrosine metabolism), purine metabolism, oxidative stress (e.g., pentose phosphate pathway (PPP) and glutathione metabolism), etc.

The related differential metabolites involved in the disturbed pathways were visualized using a pathway map (Figure 4B). As a glycolysis intermediate, 3-phosphoglyceric acid levels decreased in BCs, while lactic acid levels were higher. Reduced TCA cycle intermediates were also observed in the BC group. The reduced TCA cycle activity and active anaerobic glycolysis in BCs imply that the energy supply was converted from aerobic oxidation via the TCA cycle to anaerobic glycolysis. This finding agrees with the “Warburg effect” [22, 23] and the abnormal expression of related genes in BC [24], such as the reduction in the pyruvate dehydrogenase complex [11].

PPP intermediate levels (e.g., D-glucuronic acid, D-ribose) were decreased in the BC group. PPP provides precursor substances and reducing power for nucleotide and reductive synthesis [25, 26]. Glycine, a precursor of purine synthesis [27], was reduced in the BC group. Decreased adenine and inosine levels were also observed. These results illustrate that purine synthesis, a vital metabolism process for cell proliferation, was disturbed in BC. Cholesterol, which has important cellular functions [28] (e.g., cell signaling and cell proliferation), was increased in the BC group. The disturbed PPP, purine synthesis and cholesterol in BCs may be related to active anabolic metabolism in tumor cells.

The redox state of a cell can be revealed by the ratio of lactic acid/alanine, which has a positive correlation with oxidative stress [24]. In our study, the ratio was significantly elevated in the BC group for both the discovery (*p* < 0.001, ratio _BC/HC_ = 3.4) and external validation (*p* < 0.001, ratio _BC/HC_ = 1.7) sets. Glycine as a participant in glutathione (GSH) synthesis was decreased in the BC group. GSH is a primary cellular antioxidant that protects cells from oxidative damage [27]. The high lactic acid/alanine ratio and abnormal glycine and PPP intermediate levels in BC may be connected with high oxidative stress in tumor cells.

### Potential biomarkers for BC diagnosis

**Figure 5:**
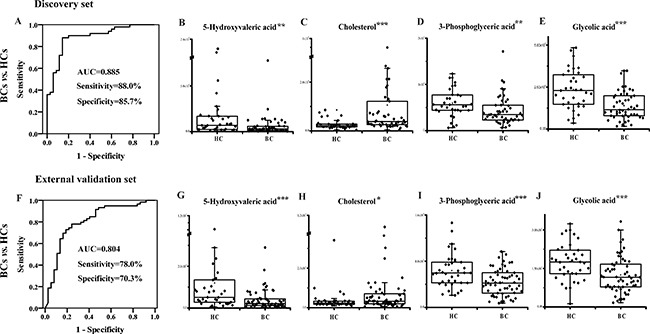
Diagnostic performance of the urinary four-biomarker panel for the diagnosis of BC ROC curves for the four-biomarker panel for BCs vs. HCs in the (**A**) discovery set and the (**F**) external validation set. Urinary concentration of (**B**) 5-hydroxyvaleric acid, (**C**) cholesterol, (**D**) 3-phosphoglyceric acid and (**E**) glycolic acid in the discovery set. Urinary concentration of (**G**) 5-hydroxyvaleric acid, (**H**) cholesterol, (**I**) 3-phosphoglyceric acid and (**J**) glycolic acid in the external validation set. *, **, and *** represent *p* values less than 0.05, 0.01, and 0.001 between BCs and HCs, respectively.

The 58 validated differential metabolites were further filtered for potential biomarker screening for BC diagnosis in the discovery set. To control the stability of the metabolite analysis, only differential metabolites with RSDs < 15% in the QC samples and an FDR < 0.05 were considered to further reduce the false discovery rate. Differential metabolites with ratios of less than 0.7 or more than 1.3 between the BC and HC groups were kept. Twenty-four candidate metabolites were used for binary logistic regression analysis performed by the SPSS 18.0 software. A four-biomarker panel (5-hydroxyvaleric acid, cholesterol, 3-phosphoglyceric acid and glycolic acid) that covered extensive metabolic characteristics (e.g., organic acid metabolism, steroid hormone biosynthesis, glycolysis and glyoxylate metabolism) was defined as a combinatorial biomarker for the discrimination of BCs vs. HCs. A receiving operator characteristic (ROC) curve was obtained using the urinary four-biomarker panel. The area under the curve (AUC) was 0.885 for BC diagnosis, with 88.0% sensitivity and 85.7% specificity (Figure 5A), showing satisfactory discrimination by the four-biomarker panel. The relative contents of 5-hydroxyvaleric acid, cholesterol, 3-phosphoglyceric acid, and glycolic acid are shown in Figure 5B–5E.

In the external validation set, the probability was calculated for ROC analysis using the binary logistic regression model established in the discovery set. The AUC was 0.804 for BC discrimination, with 78.0% sensitivity and 70.3% specificity (Figure 5F). For all of the subjects from both the discovery and validation groups, the AUC was 0.846 for BC diagnosis, with 81.7% sensitivity and 79.2% specificity. The relative contents of the four metabolites are shown in Figure 5G–5J. Among the four metabolites, cholesterol levels were significantly increased in the BC group, while 5-hydroxyvaleric acid, 3-phosphoglyceric acid, and glycolic acid levels were markedly decreased in the BC group.

**Figure 6:**
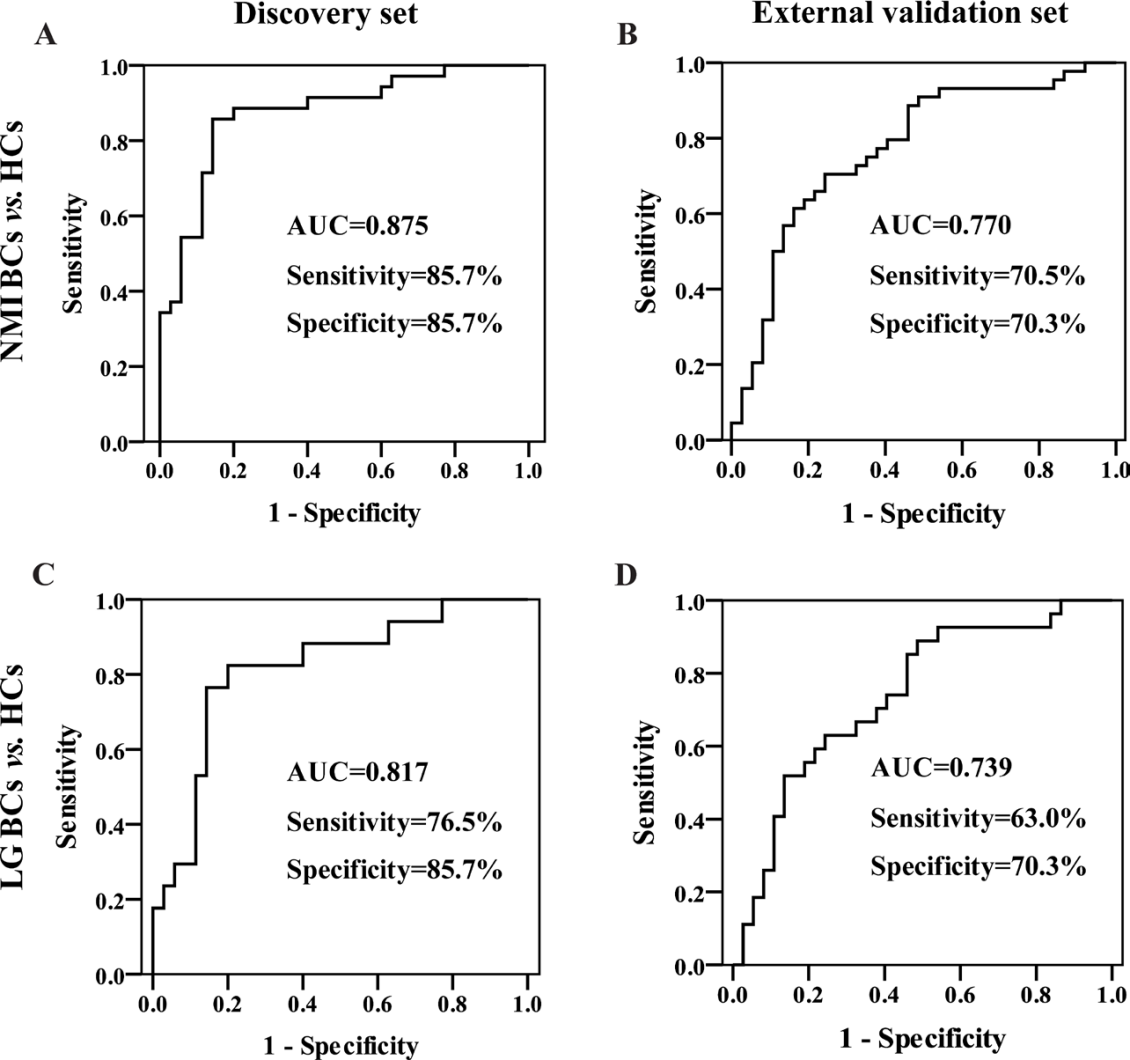
Diagnostic performance of the urinary four-biomarker panel for the diagnosis of early-stage BC ROC curves of the urinary four-biomarker panel for NMI BCs vs. HCs in the (**A**) discovery set and (**B**) external validation set. ROC curves of the urinary four-biomarker panel for LG BCs vs. HCs in the (**C**) discovery set and (**D**) external validation set.

To identify the potential ability for early-stage BC diagnosis, the urinary four-biomarker panel was applied to distinguish NMI or low-grade (LG) BCs from HCs. ROC analysis was performed based on the same binary logistic regression model for BC diagnosis. For the NMI BC diagnosis, the AUC, sensitivity, and specificity were 0.875, 85.7%, and 85.7% in the discovery set (Figure 6A) and 0.770, 70.5% and 70.3% in the external validation set (Figure 6B), respectively. For LG BC diagnosis, the AUC, sensitivity, and specificity were 0.817, 76.5%, and 85.7% in the discovery set (Figure 6C) and 0.739, 63.0% and 70.3% in the external validation set (Figure 6D), respectively. For all of the subjects from both the discovery and validation groups, the AUC was 0.825 for NMI BC diagnosis, with 77.2% sensitivity and 79.2% specificity, and 0.781, 68.2% and 79.2% for LG BC diagnosis, respectively. These results reveal that the urinary four-biomarker panel can be used for the diagnosis of NMI or LG BC.

In conclusion, a urinary GC-MS-based pseudotargeted method was developed and validated. This approach was found to be suitable for metabolic profiling analysis due to its good analytical performance. Using the developed method, the significantly differential urinary metabolites between BCs and HCs were identified and tested in two independent groups, demonstrating that energy supply, anabolic metabolism and cell redox states are disturbed in BC. A combinatorial biomarker panel consisting of four differential metabolites was defined for BC and early-stage BC diagnosis with satisfactory sensitivity and specificity in both the discovery and external validation sets. This study provides a new candidate urinary biomarker for BC (even early-stage BC) diagnosis. However, a large perspective cohort study is still needed to verify the usefulness of combinatorial biomarkers in the future.

## MATERIALS AND METHODS

### Chemicals and reagents

HPLC-grade methanol was supplied by Merck (Darmstadt, Germany). Ultrapure water was prepared with a Milli-Q system (Millipore, USA). N-methyl-N-(trimethylsilyl)-trifluoroacetamide (MSTFA), methoxyamine hydrochloride, pyridine and urease (Type 3) were obtained from Sigma-Aldrich (St. Louis, MO, USA). The chemical standards for metabolite structure validation were purchased from Sigma-Aldrich, Alfa Aesar (Ward Hill, MA, USA) or J&K Scientific (Beijing, China).

### Urine sample collection

This study was approved by the ethics committee of Shanghai Changhai Hospital, and informed consent forms were obtained from all participants. BC patients were diagnosed by histopathological examination based on the World Health Organization/International Society of Urological Pathology. Urine samples from BC patients and matched HC subjects in the discovery and external validation sets were obtained at Shanghai Changhai Hospital and Shanghai Medical Center, respectively. Subjects with hypohepatia or renal dysfunction were excluded. The fasting morning urine samples were collected and stored at –80°C.

In the discovery set, 85 urine samples were enrolled, including 50 BC subjects (male/female: 35/15) and 35 HC subjects (male/female: 23/12). The BC group contained 17 LG BC subjects, 33 HG BC subjects, 35 NMI BC subjects and 15 muscle invasive (MI) BC subjects. In the external validation set, another 96 urine samples were enrolled, including 59 BC subjects (male/female: 51/8) and 37 HC subjects (male/female: 26/11). The BC group contained 27 LG BC subjects, 32 HG BC subjects, 44 NMI BC subjects and 15 MI BC subjects. The BC and HC groups were age- and sex-matched in both the discovery and external validation sets. Clinical information is given in Table 1.

**Table 1:** Clinical information for the subjects enrolled in the discovery and external validation sets

Characteristics	Discovery set		External validation set	
	HC	BC	HC	BC
Cases	35	50	37	59
Age*	63.1 ± 8.1	62.8 ± 12.1	65.9 ± 6.0	65.0 ± 11.6
Sex (Male/Female)	23/12	35/15	26/11	51/8
Grade (Low/High)		17/33		27/32
Stage (NMI/MI)		35/15		44/15

^*^Data are given as the mean ± SD.

### Urine sample preparation

Urinary samples were thawed before preparation. One hundred microliters of urease solution (15 mg/mL in ultrapure water) was added to 100 μL of urine, vortexed for 10 s, and placed in a 37°C water bath for 15 min for enzymatic hydrolysis of the urea. Next, 800 μL of methanol was added to the above solution, which was then vortexed for 30 s. After centrifugation, 400 μL of the supernatant was lyophilized. Before analysis, 50 μL of methoxyamine solution (20 mg/mL in pyridine) was added to the residue with vortexing and ultrasound treatment and was then maintained in a 37°C water bath for 1.5 h to oximate. Next, 40 μL of MSTFA was added to the above solution, which was held in a 37°C water bath for 1 h to silanize. Finally, the supernatant was used for GC-MS analysis.

Equal volumes of all urinary samples were pooled to prepare the QC samples. The repeatability was assessed by the parallel processing of six QC samples using the same procedure employed for the original samples. Next, 4-fold, 2-fold, 1-fold, 0.5-fold and 0.25-fold concentrations of the QC samples were obtained by lyophilizing a certain volume of the QC sample and dissolving it in ultrapure water. For linearity evaluation, each concentration level of the QC samples was pretreated with three duplications using the same procedure utilized for the initial samples. The linearity was evaluated by calculating the Pearson correlation coefficient between the MS response and the metabolite concentration.

### Nontargeted GC−MS analysis

A QP 2010 GC-MS system with an AOC-20i automatic injector (Shimadzu, Japan) coupled with a DB-5 MS fused-silica capillary column (30 m × 0.25 mm × 0.25 μm, Agilent Technologies, USA) was used for GC-MS analysis. The column temperature was held at 70°C for 3 min, increased to 220°C at a rate of 4°C/min, and then increased again at a rate of 8°C /min to 300°C for 10 min. The injection temperature, transfer line and ion source were maintained at 300°C, 280°C and 230°C, respectively. One microliter of sample was injected at a split ratio of 1:10. The carrier gas, helium (99.9995%, China), was maintained at a constant linear velocity of 40 cm/s, and the electron ionization source voltage was 70 eV. Data acquisition started at 5.0 min with a mass range of 33–600 m/z.

### Establishment of a urinary pseudotargeted GC-MS metabolomics method

The procedures for transforming the nontargeted method to a pseudotargeted method were as follows [18]. First, the QC samples were analyzed using a nontargeted method. AMDIS 2.62 (NIST, USA) was employed for the QC data analysis to obtain the scan time, intensity, S/N, retention time (RT) and start and end times of the peaks. The data were pretreated by keeping the ions with the highest intensity in the 1-s scan window and with an S/N > 20. Then, characteristic ions were selected using an in-house software program with a bi-Gaussian chromatographic peak algorithm [18] and ChromTOF 4.43 (LECO, USA). The RT intervals of adjacent peaks were used for grouping. Additionally, a 3-fold volume QC sample and a hydrocarbon mixture were analyzed to annotate the low-abundance metabolites and to calculate the retention index (RI) based on n-alkanes, respectively. The identification of urinary metabolites was similar to a previous report [8]. In short, metabolites were annotated based on commercial mass spectral libraries (Mainlib, NIST, Wiley, and Fiehn) and a homemade metabolite library and were further verified using the standards’ RT/RI. The developed pseudotargeted method was applied to study BC urinary metabolic profiling using the same GC conditions as the nontargeted method and a SIM mode with an acquisition rate of 4 scans/s. A QC sample was inserted every ten samples to monitor the stability of the system while running the sequences.

### Data analysis

A peak table was exported by sequentially processing the raw data using Postrun Analysis based on a quantitative table (containing RT and characteristic ions). The peak area was normalized to the total peak area. The identified metabolites with an RSD below 30% in the QC samples were further analyzed. PCA and PLS-DA were performed using SIMCA-P 11.0 (Umetrics, Sweden). The differential metabolites with a *p* < 0.05 were screened by a nonparametric test (Mann-Whitney *U* test) using the SPSS 18.0 software. To reduce the false positive rate, FDR correction was performed using the Benjamini–Hochberg method [29]. Changes in the levels of the differential metabolites were visualized with MultiExperiment Viewer (http://www.tm4.org). The altered pathways were determined with MetaboAnalyst 2.0 (http://www.metaboanalyst.ca) and pathway maps of the differential metabolites were displayed with VANTED [30]. The potential biomarkers were defined by binary logistic regression analysis using the SPSS 18.0 software and were presented as box plots using the Origin 8.0 software.

## SUPPLEMENTARY MATERIALS FIGURES AND TABLES




