# Supplementary material for: Discovery and validation of potential urinary biomarkers for bladder cancer diagnosis using a pseudotargeted GC-MS metabolomics method
Source: Oncotarget. 2017 Feb 1;8(13):20719–28. doi: 10.18632/oncotarget.14988 (PMC5400539; doi:10.18632/oncotarget.14988)
Supplement: Supplementary file 1 [file oncotarget-08-20719-s001.pdf]

Discovery and validation of potential urinary biomarkers for bladder cancer diagnosis using a pseudotargeted GC-MS metabolomics method

Supplementary Materials

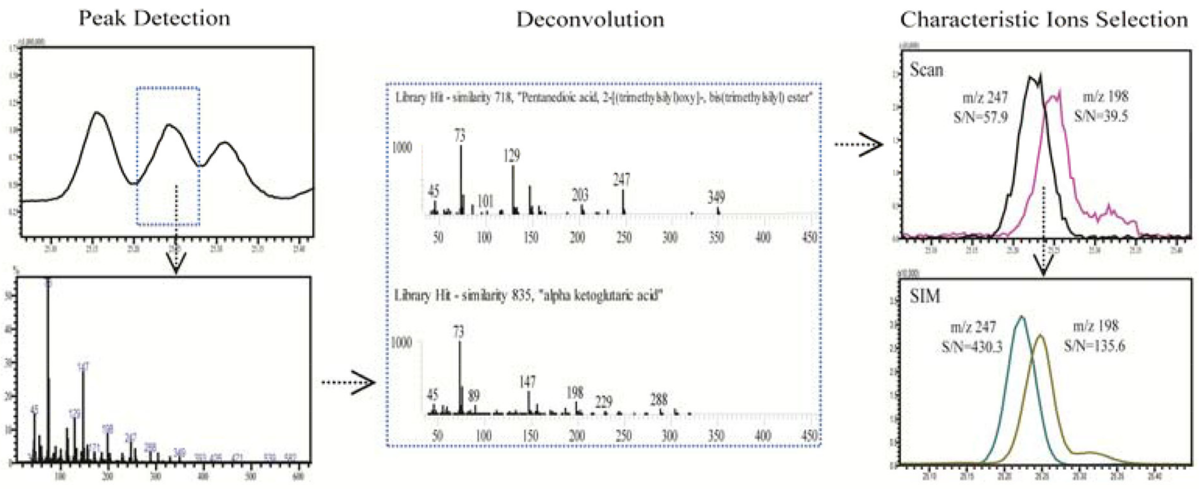

Supplementary Figure 1: An example of the characteristic ion selection.

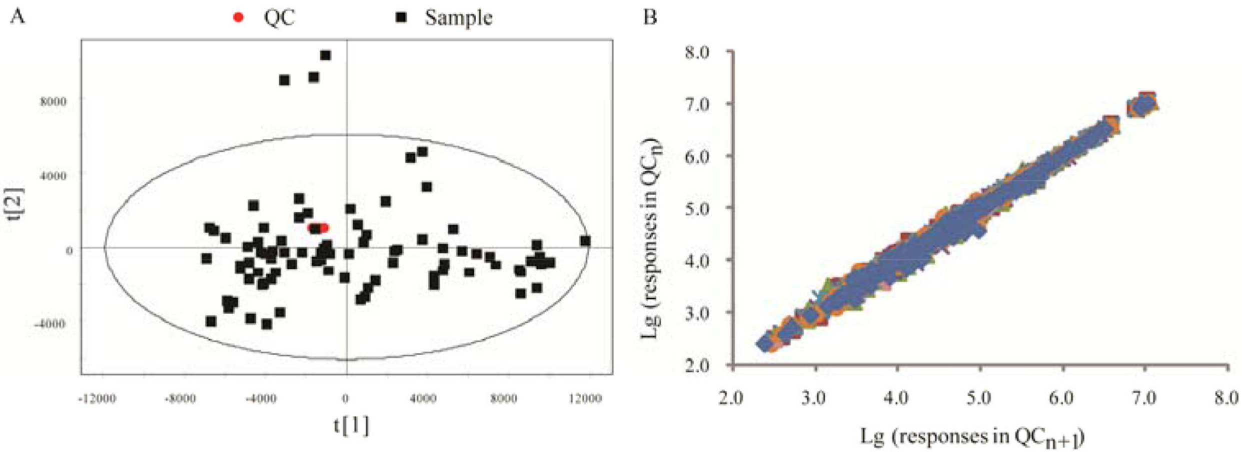

Supplementary Figure 2: Quality monitoring of the entire analysis process. (**A**) PCA score scatter plots of the samples and QCs. (**B**) Scatter plots of the peaks in QC_n and QC_{n+1} (n = 1, 2, … , 9).

Supplementary Table 1: Metabolites identified by the developed urinary pseudotargeted metabolomics method. See Supplementary_Table_1

Supplementary Table 2: Differential metabolites and related pathways in the discovery and external validation sets. See Supplementary_Table_2
