# Supplementary material for: Discovery and validation of potential urinary biomarkers for bladder cancer diagnosis using a pseudotargeted GC-MS metabolomics method
Source: Oncotarget. 2017 Feb 1;8(13):20719–28. doi: 10.18632/oncotarget.14988 (PMC5400539; doi:10.18632/oncotarget.14988)
Supplement: Supplementary file 2 [file oncotarget-08-20719-s002.doc]

**Table S1**. Metabolites identified by the developed urinary pseudotargeted metabolomics method.

| No. | Metabolites | Identification  level^a^ | RT  (min) | RI^b^ | Characteristic  ions (m/z) | Category |
| --- | --- | --- | --- | --- | --- | --- |
| 1 | Ethylamine | 2 | 5.20 | 957.5 | 174 | Amine |
| 2 | Ethylene Glycol | 2 | 6.07 | 983.1 | 147 | Alcohol |
| 3 | N,N-Dimethylglycine | 2 | 6.15 | 986.8 | 58 | Amino acid |
| 4 | 1,2-Propanediol | 2 | 6.46 | 995.5 | 117 | Alcohol |
| 5 | 2-Hydroxypyridine | 1 | 7.52 | 1030.0 | 152 | Others |
| 6 | 2,3-Butanediol | 1 | 7.67 | 1034.6 | 117 | Alcohol |
| 7 | Pyruvic acid | 1 | 7.97 | 1045.1 | 174 | Organic acid |
| 8 | Phenol | 2 | 8.06 | 1047.4 | 151 | Phenol |
| 9 | 1,3-Propanediol | 2 | 8.16 | 1050.8 | 130 | Alcohol |
| 10 | Lactic acid | 1 | 8.28 | 1054.7 | 117 | Organic acid |
| 11 | 2-Hydroxyisobutyric acid | 1 | 8.35 | 1056.8 | 205 | Organic acid |
| 12 | Hexanoic acid | 1 | 8.75 | 1069.4 | 173 | Organic acid |
| 13 | Glycolic acid | 1 | 8.83 | 1072.7 | 205 | Organic acid |
| 14 | Pyruvic acid, enol | 2 | 9.15 | 1083.1 | 147 | Organic acid |
| 15 | Valine | 1 | 9.21 | 1085.1 | 72 | Amino acid |
|  |  |  | 13.49 | 1212.1 | 144 |  |
| 16 | Alanine | 1 | 9.67 | 1098.8 | 116 | Amino acid |
|  |  |  | 18.21 | 1352.4 | 188 |  |
| 17 | Hydroxylamine | 1 | 9.95 | 1107.8 | 146 | Amine |
| 18 | Glycine | 1 | 10.19 | 1120.1 | 102 | Amino acid |
|  |  |  | 16.51 | 1300.8 | 174 |  |
| 19 | Levulinic acid | 2 | 10.37 | 1122.0 | 143 | Organic acid |
| 20 | 2-Methylacetoacetic acid | 2 | 10.47 | 1123.1 | 245 | Organic acid |
| 21 | 3-Methyl-2-Oxobutanoic acid | 1 | 10.58 | 1125.8 | 89 | Organic acid |
| 22 | 2-Hydroxy-2-Methylbutyric acid | 1 | 10.66 | 1128.8 | 145 | Organic acid |
| 23 | 3-Hydroxypyridine | 1 | 10.73 | 1131.3 | 152 | Others |
| 24 | Oxalic acid | 1 | 10.77 | 1132.1 | 147 | Organic acid |
| 25 | 3-Hydroxypropionic acid | 1 | 11.12 | 1142.5 | 177 | Organic acid |
| 26 | p-Cresol | 2 | 11.29 | 1147.5 | 165 | Phenol |
| 27 | 4-Hydroxypyridine | 1 | 11.35 | 1148.1 | 152 | Others |
| 28 | Leucine | 1 | 11.49 | 1153.3 | 86 | Amino acid |
| 29 | 3-Hydroxybutyric acid | 1 | 11.65 | 1158.1 | 233 | Organic acid |
| 30 | Isoleucine | 1 | 12.19 | 1174.1 | 86 | Amino acid |
| 31 | 3-Methyl-2-Oxovaleric acid | 1 | 12.26 | 1175.8 | 89 | Organic acid |
| 32 | 2-Methyl-3-Hydroxybutyric acid | 1 | 12.86 | 1193.6 | 117 | Organic acid |
| 33 | Mimosine | 1 | 12.92 | 1195.3 | 188 | Amino acid |
|  |  |  | 20.64 | 1427.4 | 240 |  |
| 34 | N-Acetyl-2-Aminoethanol | 2 | 13.15 | 1203.1 | 160 | Amine |
| 35 | 3-Hydroxyisovaleric acid | 1 | 13.33 | 1207.4 | 131 | Organic acid |
| 36 | Methylmalonic acid | 1 | 13.50 | 1212.5 | 147 | Organic acid |
| 37 | 2-Methoxyphenol | 2 | 13.82 | 1221.4 | 166 | Phenol |
| 38 | 2-Ethyl-3-Hydroxypropionic acid | 2 | 13.99 | 1226.8 | 247 | Organic acid |
| 39 | 4-Hydroxybutyric acid | 2 | 14.29 | 1234.7 | 233 | Organic acid |
| 40 | Benzoic acid | 1 | 14.54 | 1243.3 | 179 | Organic acid |
| 41 | 3-Octenoic acid | 2 | 14.83 | 1251.7 | 199 | Organic acid |
| 42 | Serine | 1 | 14.92 | 1254.0 | 132 | Amino acid |
|  |  |  | 18.44 | 1359.0 | 218 |  |
| 43 | Ethanolamine | 1 | 15.18 | 1261.7 | 174 | Amine |
| 44 | Phosphate | 1 | 15.48 | 1269.0 | 207 | Others |
| 45 | Glycerol | 1 | 15.59 | 1273.7 | 205 | Alcohol |
| 46 | Ethylmalonic acid | 1 | 15.74 | 1278.3 | 147 | Organic acid |
| 47 | 1,2,3-Butanetriol | 1 | 16.09 | 1288.4 | 205 | Alcohol |
| 48 | Nicotinic acid | 1 | 16.11 | 1289.6 | 180 | Organic acid |
| 49 | Threonine | 1 | 16.16 | 1290.5 | 130 | Amino acid |
|  |  |  | 19.24 | 1383.4 | 218 |  |
| 50 | 3-Hydroxyhexanoic acid | 2 | 16.65 | 1304.7 | 145 | Organic acid |
| 51 | Pyrocatechol | 1 | 16.79 | 1309.4 | 254 | Phenol |
| 52 | Succinic acid | 1 | 16.93 | 1313.5 | 247 | Organic acid |
| 53 | Picolinic acid | 2 | 17.06 | 1317.2 | 180 | Organic acid |
| 54 | Methyl succinic acid | 1 | 17.30 | 1324.6 | 261 | Organic acid |
| 55 | Glyceric acid | 1 | 17.44 | 1328.7 | 292 | Organic acid |
| 56 | 1,2-Butanediol | 2 | 17.47 | 1329.6 | 131 | Alcohol |
| 57 | 5-Hydroxyvaleric acid | 2 | 17.56 | 1332.5 | 247 | Organic acid |
| 58 | Uracil | 1 | 17.61 | 1333.8 | 241 | Nucleoside and derivative |
| 59 | (R,S)-2,3-Dihydroxybutanoic acid | 2 | 17.83 | 1340.5 | 292 | Organic acid |
| 60 | (R,R)-2,3-Dihydroxybutanoic acid | 2 | 18.14 | 1349.9 | 292 | Organic acid |
| 61 | Fumaric acid | 1 | 18.17 | 1351.0 | 245 | Organic acid |
| 62 | 5-Hydroxyhexanoic acid | 2 | 18.31 | 1355.2 | 117 | Organic acid |
| 63 | Nonanoic acid | 1 | 18.47 | 1359.9 | 215 | Organic acid |
| 64 | 1,2,4-Butanetriol | 2 | 18.52 | 1361.0 | 103 | Alcohol |
| 65 | Sarcosine | 1 | 19.04 | 1377.0 | 116 | Amino acid |
| 66 | 4-Methylcatechol | 2 | 19.23 | 1383.0 | 268 | Phenol |
| 67 | 2,3-Dihydroxyisovaleric acid | 1 | 19.42 | 1388.6 | 131 | Organic acid |
| 68 | Hydroquinone | 1 | 19.71 | 1397.7 | 239 | Phenol |
| 69 | Methionine | 1 | 19.96 | 1405.0 | 104 | Amino acid |
| 70 | Pentanedioic acid | 1 | 19.96 | 1405.6 | 261 | Organic acid |
| 71 | 2,4-Dihydroxybutanoic acid | 2 | 20.15 | 1411.1 | 103 | Organic acid |
| 72 | Beta-Alanine | 1 | 20.54 | 1423.6 | 248 | Amino acid |
| 73 | 3-Methylglutaric acid | 2 | 20.58 | 1424.7 | 69 | Organic acid |
| 74 | 3,4-Dihydroxybutanoic acid | 2 | 20.83 | 1432.8 | 233 | Organic acid |
| 75 | 3-Methyl-2-Pentenedioic acid | 2 | 21.00 | 1438.3 | 109 | Organic acid |
|  |  |  | 22.42 | 1483.2 | 231 |  |
| 76 | 3-Aminoisobutyric acid | 1 | 21.42 | 1451.7 | 174 | Amino acid |
| 77 | Iminodiacetic acid | 2 | 21.87 | 1465.9 | 232 | Amino acid |
| 78 | Aminomalonic acid | 1 | 21.90 | 1466.6 | 218 | Amino acid |
| 79 | 4-Pentenoic acid | 2 | 22.21 | 1476.8 | 157 | Organic acid |
| 80 | Malic acid | 1 | 22.56 | 1487.7 | 233 | Organic acid |
| 81 | 2-Pyrrolidone-5-Carboxylic acid | 2 | 22.75 | 1489.0 | 84 | Organic acid |
| 82 | 4-Acetamidobutyric acid | 2 | 22.82 | 1495.8 | 86 | Amino acid |
| 83 | D-Threitol | 1 | 22.94 | 1499.4 | 217 | Saccharide and derivative |
| 84 | Meso-Erythritol | 1 | 23.16 | 1506.9 | 217 | Saccharide and derivative |
| 85 | 4-Hydroxycyclohexanecarboxylic acid | 2 | 23.18 | 1508.1 | 273 | Organic acid |
| 86 | Glutamic acid | 1 | 23.37 | 1514.1 | 156 | Amino acid |
|  |  |  | 23.87 | 1530.6 | 84 |  |
|  |  |  | 26.48 | 1619.0 | 246 |  |
| 87 | 2-Aminooctanoic acid | 2 | 23.40 | 1515.3 | 186 | Amino acid |
| 88 | Cytosine | 1 | 23.42 | 1515.8 | 254 | Nucleoside and derivative |
| 89 | Aspartic acid | 1 | 23.51 | 1519.0 | 232 | Amino acid |
| 90 | 4-Aminobutyric acid | 1 | 23.69 | 1525.2 | 174 | Amino acid |
| 91 | 1-Deoxy-Pentitol | 2 | 23.88 | 1531.1 | 117 | Saccharide and derivative |
| 92 | Pyrogallol | 2 | 23.89 | 1531.6 | 239 | Phenol |
| 93 | Phenylalanine | 1 | 24.12 | 1539.2 | 120 | Amino acid |
|  |  |  | 26.46 | 1618.5 | 192 |  |
| 94 | Erythronic acid | 2 | 24.19 | 1541.6 | 292 | Saccharide and derivative |
| 95 | Creatinine | 1 | 24.29 | 1545.2 | 329 | Amino acid |
| 96 | 2-Methyl-2,3-Dihydroxypropanoic acid | 2 | 24.39 | 1548.3 | 219 | Organic acid |
| 97 | 5-Hydroxymethyl-Furan-2-Carboxylic acid | 2 | 24.41 | 1548.7 | 123 | Organic acid |
| 98 | Cysteine | 1 | 24.48 | 1551.4 | 218 | Amino acid |
| 99 | L-Threonic acid | 1 | 24.74 | 1559.8 | 292 | Saccharide and derivative |
| 100 | L-Dithiothreitol | 1 | 25.14 | 1573.8 | 116 | Saccharide and derivative |
| 101 | 2-Hydroxyglutaric acid | 1 | 25.20 | 1575.2 | 247 | Organic acid |
| 102 | Alpha-Ketoglutaric acid | 1 | 25.23 | 1576.1 | 198 | Organic acid |
| 103 | 3-Hydroxyglutaric Acid | 1 | 25.24 | 1576.8 | 185 | Organic acid |
| 104 | Proline | 1 | 25.25 | 1577.2 | 142 | Amino acid |
| 105 | 1,2-Benzenedicarboxylic acid diethyl ester | 2 | 25.46 | 1584.3 | 149 | Ester |
| 106 | Hypotaurine | 1 | 25.65 | 1590.7 | 188 | Amino acid |
| 107 | 3-Hydroxy-3-Methylglutaric acid | 1 | 25.92 | 1599.3 | 247 | Organic acid |
| 108 | 3-Hydroxyphenylacetic acid | 1 | 26.04 | 1603.6 | 164 | Organic acid |
| 109 | 4-Hydroxybenzoic acid | 1 | 26.59 | 1623.0 | 267 | Organic acid |
| 110 | 2-Aminoadipic acid | 2 | 26.71 | 1627.3 | 98 | Amino acid |
| 111 | 4-Hydroxyphenylacetic acid | 1 | 26.94 | 1635.4 | 252 | Organic acid |
| 112 | Tartaric acid | 1 | 26.97 | 1636.2 | 292 | Saccharide and derivative |
| 113 | 2-p-Hydroxyphenylpropionate | 2 | 27.07 | 1639.7 | 193 | Organic acid |
| 114 | D-Lyxose | 1 | 27.19 | 1644.0 | 307 | Saccharide and derivative |
|  |  |  | 27.69 | 1661.1 | 103 |  |
| 115 | 2,5-Furandicarboxylic acid | 2 | 27.22 | 1645.1 | 285 | Organic acid |
| 116 | N-Acetyl-Aspartic acid | 1 | 27.45 | 1652.9 | 158 | Amino acid |
| 117 | D-Xylose | 1 | 27.49 | 1654.3 | 103 | Saccharide and derivative |
| 118 | Pyrophosphate | 1 | 27.54 | 1656.4 | 451 | Others |
| 119 | 2-Deoxy-Ribonic acid | 2 | 27.59 | 1657.7 | 335 | Saccharide and derivative |
| 120 | D-Lyxosylamine | 1 | 27.61 | 1658.5 | 103 | Saccharide and derivative |
| 121 | 2-Hydroxyhexanedioic acid | 2 | 27.91 | 1669.4 | 261 | Organic acid |
| 122 | Xylulose | 2 | 28.04 | 1673.6 | 205 | Saccharide and derivative |
| 123 | D-Ribose | 1 | 28.10 | 1675.5 | 103 | Saccharide and derivative |
| 124 | 3-Hydroxyhexanedioic acid | 2 | 28.27 | 1681.9 | 363 | Organic acid |
| 125 | 2-Amino-4,6-Dihydroxypyrimidine | 2 | 28.34 | 1684.6 | 328 | Nucleoside and derivative |
| 126 | Anthranilic acid | 2 | 28.37 | 1685.8 | 119 | Amino acid |
| 127 | Xylitol | 1 | 28.56 | 1692.1 | 307 | Saccharide and derivative |
|  |  |  | 28.84 | 1701.7 | 217 |  |
| 128 | Levoglucosan | 1 | 28.68 | 1696.4 | 204 | Saccharide and derivative |
| 129 | D-Rhamnose | 1 | 29.11 | 1712.0 | 117 | Saccharide and derivative |
|  |  |  | 29.29 | 1718.6 | 160 |  |
| 130 | Arabitol | 1 | 29.23 | 1716.4 | 217 | Saccharide and derivative |
| 131 | Ribitol | 1 | 29.35 | 1720.6 | 217 | Saccharide and derivative |
| 132 | L-Fucose | 1 | 29.62 | 1730.7 | 117 | Saccharide and derivative |
| 133 | Citrulline | 1 | 29.82 | 1738.4 | 184 | Amino acid |
|  |  |  | 32.05 | 1821.0 | 256 |  |
| 134 | Orotic acid | 1 | 29.93 | 1742.4 | 254 | Nucleoside and derivative |
| 135 | Ornithine | 1 | 29.97 | 1744.3 | 174 | Amino acid |
|  |  |  | 31.78 | 1811.1 | 142 |  |
| 136 | Cis-Aconitic acid | 1 | 30.14 | 1749.7 | 229 | Organic acid |
| 137 | 2-Keto-Gluconic acid | 2 | 30.38 | 1759.0 | 292 | Saccharide and derivative |
| 138 | Glycerol 3-Phosphate | 1 | 30.41 | 1759.9 | 357 | Ester |
| 139 | 6-Deoxy-D-Glucitol | 2 | 30.54 | 1765.0 | 117 | Saccharide and derivative |
| 140 | Homovanillic acid | 1 | 30.56 | 1765.5 | 326 | Organic acid |
| 141 | Ribonic acid | 2 | 30.64 | 1768.0 | 292 | Saccharide and derivative |
|  |  |  | 30.86 | 1776.5 | 292 |  |
| 142 | 4-Hydroxymandelic acid | 2 | 30.87 | 1777.0 | 267 | Organic acid |
| 143 | N-Acetyl-Glutamic acid | 1 | 30.93 | 1779.2 | 156 | Amino acid |
| 144 | Azelaic acid | 1 | 31.36 | 1795.2 | 317 | Organic acid |
| 145 | Hypoxanthine | 1 | 31.39 | 1796.8 | 265 | Nucleoside and derivative |
| 146 | 3-Phosphoglyceric acid | 1 | 31.60 | 1804.7 | 357 | Organic acid |
| 147 | D-Fructofuranose | 2 | 31.69 | 1807.3 | 217 | Saccharide and derivative |
| 148 | Citric acid | 1 | 31.92 | 1816.4 | 273 | Organic acid |
| 149 | Isocitric acid | 1 | 32.03 | 1820.6 | 245 | Organic acid |
| 150 | D-Pinitol | 2 | 32.07 | 1822.5 | 260 | Saccharide and derivative |
|  |  |  | 35.27 | 1948.7 | 260 |  |
| 151 | (4-Hydroxy-3-Methoxyphenyl)ethylene glycol | 2 | 32.25 | 1829.9 | 297 | Alcohol |
| 152 | Methylcitric acid | 2 | 32.29 | 1830.9 | 287 | Organic acid |
| 153 | Hippuric acid | 1 | 32.39 | 1835.2 | 206 | Organic acid |
| 154 | Lysine | 1 | 32.66 | 1845.4 | 200 | Amino acid |
| 155 | 1,5-Anhydroglucitol | 1 | 32.66 | 1845.4 | 217 | Saccharide and derivative |
| 156 | 3-(3-Hydroxyphenyl)-3-hydroxypropionic acid | 2 | 32.70 | 1846.8 | 267 | Organic acid |
| 157 | Myristic acid | 1 | 32.70 | 1847.2 | 285 | Fatty acid |
| 158 | D-Tagatose | 1 | 32.83 | 1851.7 | 103 | Saccharide and derivative |
| 159 | Quinic acid | 1 | 32.85 | 1852.6 | 345 | Organic acid |
| 160 | Adenine | 1 | 32.94 | 1856.5 | 264 | Nucleoside and derivative |
| 161 | Sorbose | 1 | 33.13 | 1863.3 | 103 | Saccharide and derivative |
| 162 | D-Fructose | 1 | 33.22 | 1866.7 | 103 | Saccharide and derivative |
|  |  |  | 33.46 | 1876.5 | 103 |  |
| 163 | 2-O-Methyl-Ascorbic acid | 2 | 33.31 | 1870.7 | 274 | Organic acid |
| 164 | D-Allose | 1 | 33.36 | 1872.5 | 319 | Saccharide and derivative |
| 165 | 4-Hydroxy-3-Methoxymandelic acid | 1 | 33.42 | 1874.7 | 297 | Organic acid |
| 166 | Tyrosine | 1 | 33.48 | 1877.4 | 179 | Amino acid |
|  |  |  | 34.85 | 1932.0 | 280 |  |
| 167 | Ethyl-D-Glucopyranoside | 2 | 33.51 | 1878.0 | 204 | Saccharide and derivative |
|  |  |  | 34.84 | 1931.6 | 204 |  |
| 168 | D-Gluconic acid lactone | 1 | 33.54 | 1879.1 | 319 | Saccharide and derivative |
| 169 | D-Galactose | 1 | 33.65 | 1883.6 | 319 | Saccharide and derivative |
|  |  |  | 33.99 | 1896.4 | 204 |  |
| 170 | N-Carbamyl-Glutamic acid | 2 | 33.72 | 1886.3 | 257 | Amino acid |
| 171 | 3-(4-Hydroxy-3-Methoxyphenyl)propionic acid | 2 | 33.74 | 1887.3 | 340 | Organic acid |
| 172 | D-Glucose | 1 | 33.85 | 1891.1 | 129 | Saccharide and derivative |
|  |  |  | 34.27 | 1908.0 | 129 |  |
| 173 | Tyramine | 1 | 34.14 | 1903.2 | 174 | Amine |
| 174 | D-Mannitol | 1 | 34.60 | 1921.6 | 319 | Saccharide and derivative |
| 175 | D-Glucuronic acid | 1 | 34.74 | 1927.0 | 333 | Saccharide and derivative |
|  |  |  | 35.08 | 1941.0 | 333 |  |
| 176 | D-Sorbitol | 1 | 34.77 | 1928.3 | 319 | Saccharide and derivative |
| 177 | Galactitol | 1 | 34.89 | 1933.2 | 217 | Saccharide and derivative |
| 178 | 3,4-Dihydroxyhydrocinnamic acid | 2 | 34.99 | 1937.6 | 398 | Organic acid |
| 179 | Ascorbic acid | 1 | 35.05 | 1940.1 | 332 | Organic acid |
| 180 | D-Chiro-Inositol | 1 | 35.21 | 1946.5 | 318 | Saccharide and derivative |
| 181 | 1H-Indole-3-Acetic acid | 1 | 35.32 | 1951.3 | 202 | Organic acid |
| 182 | Ethylgonendione | 2 | 35.36 | 1953.3 | 358 | Steroids |
| 183 | Galacturonic acid | 1 | 35.49 | 1958.1 | 333 | Saccharide and derivative |
| 184 | Pantothenic acid | 1 | 36.15 | 1984.5 | 291 | Organic acid |
| 185 | Gluconic acid | 1 | 36.21 | 1987.2 | 292 | Saccharide and derivative |
| 186 | Galactonic acid | 1 | 36.29 | 1990.3 | 292 | Saccharide and derivative |
| 187 | Mucic acid | 1 | 36.51 | 1999.2 | 333 | Saccharide and derivative |
| 188 | Guanidinosuccinic acid | 2 | 36.90 | 2016.0 | 444 | Amino acid |
| 189 | Allo-inositol | 2 | 36.99 | 2019.7 | 318 | Saccharide and derivative |
| 190 | D-Glucopyranuronic acid | 2 | 37.03 | 2021.2 | 292 | Saccharide and derivative |
| 191 | Palmitic acid | 1 | 37.56 | 2044.1 | 313 | Fatty acid |
| 192 | 2-Hydroxyhippuric acid | 2 | 37.85 | 2056.3 | 324 | Organic acid |
| 193 | Kynurenic acid | 1 | 37.85 | 2056.6 | 318 | Amino acid |
| 194 | N-Acetyl-D-Glucosamine | 1 | 38.02 | 2063.4 | 95 | Saccharide and derivative |
|  |  |  | 38.49 | 2083.2 | 319 |  |
| 195 | N-Acetyl-D-Mannosamine | 1 | 38.16 | 2069.5 | 319 | Saccharide and derivative |
|  |  |  | 38.67 | 2091.0 | 319 |  |
| 196 | N-Acetyl-D-Galactosaminitol | 2 | 38.30 | 2075.4 | 246 | Saccharide and derivative |
| 197 | 2,5-Dihydroxyindole | 2 | 38.31 | 2076.3 | 365 | Phenol |
| 198 | Myo-Inositol | 1 | 38.38 | 2079.1 | 305 | Saccharide and derivative |
| 199 | Ferulic acid | 2 | 38.53 | 2085.6 | 338 | Organic acid |
| 200 | N-Acetyl-Glucosylamine | 2 | 38.59 | 2088.3 | 173 | Saccharide and derivative |
| 201 | Uric acid | 1 | 38.79 | 2096.4 | 441 | Nucleoside and derivative |
|  |  |  | 39.83 | 2143.5 | 369 |  |
| 202 | 3-Hydroxyhippuric acid | 2 | 39.76 | 2140.3 | 294 | Organic acid |
| 203 | Isoxanthopterin | 2 | 40.50 | 2174.0 | 380 | Nucleoside and derivative |
| 204 | Tryptophan | 1 | 40.82 | 2187.9 | 202 | Amino acid |
| 205 | 5-Hydroxyindole-3-Acetic acid | 1 | 41.23 | 2207.1 | 290 | Organic acid |
| 206 | 4-Hydroxyhippuric acid | 2 | 41.46 | 2219.3 | 193 | Organic acid |
| 207 | Stearic acid | 1 | 41.90 | 2243.8 | 341 | Fatty acid |
| 208 | Xanthurenic acid | 1 | 41.99 | 2248.8 | 406 | Organic acid |
| 209 | Glyceryl-Glycoside | 2 | 43.09 | 2310.0 | 204 | Saccharide and derivative |
|  |  |  | 43.79 | 2356.1 | 204 |  |
| 210 | Uridine | 1 | 43.41 | 2331.4 | 217 | Nucleoside and derivative |
| 211 | D-Cellobiose | 1 | 43.85 | 2359.9 | 204 | Saccharide and derivative |
| 212 | D-Glycero-D-gulo-Heptose | 2 | 43.99 | 2369.4 | 307 | Saccharide and derivative |
| 213 | p-Tolyl-Beta-D-Glucuronide | 2 | 44.73 | 2421.1 | 375 | Saccharide and derivative |
| 214 | 5-Methyluridine | 2 | 44.91 | 2435.7 | 217 | Nucleoside and derivative |
| 215 | Melibiose | 1 | 46.12 | 2528.1 | 204 | Saccharide and derivative |
|  |  |  | 49.59 | 2858.7 | 361 |  |
| 216 | Inosine | 1 | 46.68 | 2577.3 | 230 | Nucleoside and derivative |
| 217 | Glycerol 1-Palmitate | 1 | 46.76 | 2583.9 | 371 | Ester |
| 218 | N-Acetylneuraminic acid | 1 | 47.06 | 2610.2 | 246 | Amino acid |
| 219 | Lactulose | 2 | 47.11 | 2613.9 | 347 | Saccharide and derivative |
|  |  |  | 47.85 | 2682.9 | 361 |  |
| 220 | Sucrose | 1 | 47.23 | 2625.4 | 361 | Saccharide and derivative |
| 221 | Lactose | 1 | 47.78 | 2676.1 | 361 | Saccharide and derivative |
|  |  |  | 47.95 | 2691.8 | 361 |  |
| 222 | Maltose | 1 | 48.26 | 2722.8 | 361 | Saccharide and derivative |
|  |  |  | 48.54 | 2750.0 | 191 |  |
| 223 | D-Trehalose | 1 | 48.33 | 2730.0 | 361 | Saccharide and derivative |
| 224 | Isomaltose | 2 | 48.73 | 2769.5 | 361 | Saccharide and derivative |
| 225 | Monostearin | 1 | 48.80 | 2775.8 | 399 | Ester |
| 226 | N,N-Dimethylguanosine | 2 | 49.10 | 2806.0 | 280 | Nucleoside and derivative |
| 227 | Maltitol | 1 | 49.24 | 2820.9 | 361 | Saccharide and derivative |
| 228 | 1-Methylinosine | 2 | 50.65 | 2974.9 | 259 | Nucleoside and derivative |
| 229 | Acetaminophen glucuronide | 1 | 50.87 | 2997.7 | 375 | Saccharide and derivative |
| 230 | Cholesterol | 1 | 52.34 | 3146.8 | 329 | Steroids |
| 231 | Maltotriose | 2 | 55.19 | 3428.6 | 204 | Saccharide and derivative |

^1^ Metabolite is validated using the reference standard. ^2^ Metabolite is annotated using a mass spectral library search. d

^a^ Identification level is determined by the Metabolomics Standards Initiative (MSI).

^b^ RI is calculated based on n-alkanes.

**Table S2.** Differential metabolites and related pathways in the discovery and external validation sets.

| **Metabolites** | **Related pathways** | **Discovery set** | | |  | **External validation set** | | |
| --- | --- | --- | --- | --- | --- | --- | --- | --- |
|  |  | ***p*** | **Ratio^b^** | **FDR** |  | ***p*** | **Ratio^b^** | **FDR** |
| **Carbohydrate Metabolism Related** | |  |  |  |  |  |  |  |
| Lactic acid^a^ | Glycolysis, Pyruvate metabolism | 0.003 | 1.5 | 0.026 |  | 0.020 | 1.4 | 0.043 |
| 3-Phosphoglyceric acid^a^ | Glycolysis | 0.002 | 0.6 | 0.022 |  | <0.001 | 0.7 | 0.006 |
| Pyruvic acid, enol | Pyruvate metabolism | 0.010 | 0.6 | 0.050 |  | <0.001 | 0.5 | <0.001 |
| D-Ribose^a^ | Pentose phosphate pathway | 0.013 | 0.8 | 0.060 |  | 0.002 | 0.9 | 0.009 |
| D-Glucuronic acid^a^ | Pentose and glucuronate interconversions | 0.012 | 0.8 | 0.057 |  | 0.012 | 0.8 | 0.029 |
| D-Lyxose^a^ | Pentose and glucuronate interconversions | 0.003 | 0.8 | 0.026 |  | 0.002 | 0.8 | 0.009 |
| D-Xylose^a^ | Pentose and glucuronate interconversions | 0.012 | 0.7 | 0.057 |  | 0.002 | 0.8 | 0.009 |
| Ribitol^a^ | Pentose and glucuronate interconversions | 0.018 | 0.8 | 0.074 |  | <0.001 | 0.8 | <0.001 |
| Xylitol^a^ | Pentose and glucuronate interconversions | 0.002 | 0.7 | 0.022 |  | 0.003 | 0.9 | 0.012 |
| Xylulose | Pentose and glucuronate interconversions | 0.010 | 0.7 | 0.050 |  | 0.009 | 0.9 | 0.025 |
| D-Cellobiose^a^ | Starch and sucrose metabolism | 0.031 | 0.7 | 0.099 |  | 0.040 | 0.9 | 0.072 |
| D-Rhamnose^a^ | Fructose and mannose metabolism | 0.033 | 0.8 | 0.103 |  | <0.001 | 0.7 | <0.001 |
| L-Fucose^a^ | Fructose and mannose metabolism | 0.016 | 0.7 | 0.067 |  | <0.001 | 0.7 | <0.001 |
| D-Allose^a^ | Fructose and mannose metabolism | 0.004 | 0.8 | 0.032 |  | 0.014 | 0.9 | 0.032 |
| D-Fructofuranose | Fructose and mannose metabolism | 0.003 | 0.7 | 0.026 |  | 0.033 | 0.8 | 0.063 |
| D-Sorbitol^a^ | Fructose and mannose metabolism, Galactose metabolism | 0.004 | 0.7 | 0.032 |  | 0.002 | 0.8 | 0.009 |
| Glyceric acid^a^ | Glycerolipid metabolism, Pentose phosphate pathway | 0.045 | 0.8 | 0.129 |  | 0.009 | 0.7 | 0.025 |
| Glycerol 3-Phosphate^a^ | Glycerolipid metabolism, Glycerophospholipid metabolism | 0.001 | 0.5 | 0.015 |  | 0.002 | 0.7 | 0.009 |
| 1,3-Propanediol | Glycerolipid metabolism | 0.015 | 1.2 | 0.067 |  | 0.025 | 1.1 | 0.052 |
| Ethanolamine^a^ | Glycerophospholipid metabolism | 0.006 | 0.7 | 0.040 |  | 0.011 | 0.8 | 0.028 |
| N-Acetyl-2-Aminoethanol | Glycerophospholipid metabolism | 0.010 | 0.3 | 0.050 |  | 0.040 | 0.8 | 0.072 |
| N-Acetyl-D-Mannosamine^a^ | Amino sugar and nucleotide sugar metabolism | 0.023 | 0.8 | 0.086 |  | 0.008 | 0.9 | 0.024 |
| 1-Deoxy-Pentitol | Pentose metabolism | 0.021 | 0.7 | 0.083 |  | 0.011 | 0.8 | 0.028 |
| Glycolic acid^a^ | Glyoxylate metabolism | <0.001 | 0.5 | <0.001 |  | <0.001 | 0.7 | 0.006 |
| p-Tolyl-Beta-D-Glucuronide | Carbohydrate metabolism | 0.024 | 0.4 | 0.089 |  | 0.011 | 0.4 | 0.028 |
| Glyceryl-Glycoside | Carbohydrate metabolism | 0.026 | 0.6 | 0.093 |  | 0.026 | 0.7 | 0.053 |
| **Tricarboxylic Acid (TCA) Cycle** | |  |  |  |  |  |  |  |
| Isocitric acid^a^ | TCA cycle | 0.008 | 0.8 | 0.045 |  | 0.002 | 0.8 | 0.009 |
| cis-Aconitic acid^a^ | TCA cycle | <0.001 | 0.6 | <0.001 |  | <0.001 | 0.7 | <0.001 |
| Succinic acid^a^ | TCA cycle | 0.028 | 0.8 | 0.095 |  | <0.001 | 0.6 | <0.001 |
| **Amino Acids Metabolism** |  |  |  |  |  |  |  |  |
| N-Acetyl-Aspartic acid^a^ | Alanine, aspartate and glutamate metabolism | <0.001 | 0.6 | <0.001 |  | <0.001 | 0.7 | <0.001 |
| N-Acetyl-Glutamic acid^a^ | Alanine, aspartate and glutamate metabolism | <0.001 | 0.6 | <0.001 |  | <0.001 | 0.8 | 0.006 |
| 3-Hydroxy-3-Methylglutaric acid^a^ | Leucine metabolilsm | 0.022 | 0.8 | 0.084 |  | 0.002 | 0.8 | 0.009 |
| 3-Hydroxyisovaleric acid^a^ | Leucine metabolilsm | 0.005 | 0.7 | 0.035 |  | 0.004 | 0.8 | 0.015 |
| 3-Methyl-2-Pentenedioic acid | Leucine metabolilsm | <0.001 | 0.6 | <0.001 |  | <0.001 | 0.7 | <0.001 |
| 2-Methyl-3-Hydroxybutyric acid^a^ | Isoleucine metabolism | 0.002 | 0.7 | 0.022 |  | 0.003 | 0.7 | 0.012 |
| 3-Hydroxyphenylacetic acid^a^ | Tyrosine metabolism, Phenylalanine metabolism | 0.007 | 0.6 | 0.042 |  | <0.001 | 0.5 | <0.001 |
| Tyrosine^a^ | Tyrosine metabolism | 0.021 | 0.5 | 0.083 |  | 0.028 | 0.7 | 0.056 |
| Tyramine^a^ | Tyrosine metabolism | 0.006 | 0.6 | 0.040 |  | 0.028 | 0.7 | 0.056 |
| 4-Hydroxymandelic acid | Tyramine metabolism | 0.008 | 0.7 | 0.045 |  | 0.003 | 0.8 | 0.012 |
| Glycine^a^ | Glycine, serine and threonine metabolism | 0.005 | 0.5 | 0.035 |  | 0.007 | 0.6 | 0.022 |
| Lysine^a^ | Lysine metabolism | 0.007 | 0.3 | 0.042 |  | 0.016 | 0.6 | 0.036 |
| 2-Aminoadipic acid | Lysine metabolism | 0.027 | 0.4 | 0.095 |  | 0.003 | 0.5 | 0.012 |
| Aminomalonic acid^a^ | Amino Acids Metabolism | 0.001 | 0.6 | 0.015 |  | <0.001 | 0.5 | <0.001 |
| **Organic Acid Metabolism** |  |  |  |  |  |  |  |  |
| 2-Hydroxyglutaric acid^a^ | C5-Branched dibasic acid metabolism | 0.032 | 0.8 | 0.101 |  | <0.001 | 0.6 | <0.001 |
| 3-Hydroxypropionic acid^a^ | Organic acid metabolism | 0.001 | 0.7 | 0.015 |  | 0.029 | 0.6 | 0.057 |
| 5-Hydroxyvaleric acid | Organic acid metabolism | 0.003 | 0.4 | 0.026 |  | <0.001 | 0.4 | <0.001 |
| 5-Hydroxyhexanoic acid | Organic acid metabolism | 0.001 | 0.6 | 0.015 |  | 0.007 | 0.7 | 0.022 |
| (R,S)-2,3-Dihydroxybutanoic acid | Organic acid metabolism | 0.015 | 0.8 | 0.067 |  | 0.017 | 0.7 | 0.037 |
| 2,4-Dihydroxybutanoic acid | Organic acid metabolism | 0.029 | 0.8 | 0.096 |  | 0.011 | 0.8 | 0.028 |
| 3,4-Dihydroxybutanoic acid | Organic acid metabolism | 0.007 | 0.8 | 0.042 |  | 0.048 | 0.8 | 0.083 |
| **Nucleotide metabolism and others** | |  |  |  |  |  |  |  |
| Adenine^a^ | Purine metabolism | 0.031 | 0.3 | 0.099 |  | <0.001 | 0.5 | <0.001 |
| Inosine^a^ | Purine metabolism | 0.002 | 0.6 | 0.022 |  | <0.001 | 0.7 | <0.001 |
| Cholesterol^a^ | Steroid hormone biosynthesis | <0.001 | 1.8 | <0.001 |  | 0.043 | 1.4 | 0.076 |
| Hippuric acid^a^ | Intestinal flora metabolism | 0.016 | 0.5 | 0.067 |  | 0.038 | 0.7 | 0.070 |
| p-Cresol | Intestinal flora metabolism | 0.042 | 0.4 | 0.125 |  | 0.013 | 0.4 | 0.030 |
| 1,2,3-Butanetriol^a^ | —— | 0.029 | 0.7 | 0.096 |  | 0.007 | 0.9 | 0.022 |
| 1,2,4-Butanetriol | —— | <0.001 | 0.6 | <0.001 |  | 0.001 | 0.7 | 0.006 |
| Phosphate^a^ | —— | <0.001 | 2.6 | <0.001 |  | <0.001 | 1.7 | <0.001 |

^a^ Metabolites are confirmed by standards.

^b^ Ratios are calculated as the median of BCs/HCs.
